# Age at menarche in Korean adolescents: trends and influencing factors

**DOI:** 10.1186/s12978-016-0240-y

**Published:** 2016-09-23

**Authors:** Mee-Hwa Lee, Shin Hye Kim, Minkyung Oh, Kuk-Wha Lee, Mi-Jung Park

**Affiliations:** 1 Department of Obstetrics and Gynecology, CHA Bundang Medical Center, School of Medicine, CHA University, 59 Yatap-ro, Bundang-gu, Seongnam, 13496 Korea; 2 Department of Pediatrics, Sanggye Paik Hospital, Inje University College of Medicine, 761-1 Sanggye-7-dong, Nowon-gu, 139-707 Seoul Korea; 3 Clinical Trial Center, Busan Paik Hospital and Department of Pharmacology, Inje University College of Medicine, Busan, Korea; 4 Adventist Health System, Orlando, FL USA

**Keywords:** Menarche, Puberty, Secular trend, Adolescents

## Abstract

**Background:**

An increased incidence of central precocious puberty has been recently reported in South Korea, which suggests an ongoing downward trend in pubertal development in the Korean population. We aimed to verify the trend in age at menarche in young Korean women during the last decade and associated factors.

**Methods:**

We analyzed a population-based sample of 3409 Korean girls, aged 10–18 years, using data from the Korean National Health and Nutrition Examination Surveys (KNHANES) II (2001), III (2005), IV (2007–2009), and V (2010 and 2011). Average age at menarche was studied using the Kaplan-Meier survival method and predictors were analyzed using Cox proportional hazards model. The percentage of subjects who had experienced menarche at each age level was compared by using the Cochran-Armitage test.

**Results:**

Overall mean age at menarche was 12.7 years. The percentage of subjects who experienced menarche before the age of 12 years was 21.4 % in 2001 but increased to 34.6 % in 2010/2011 (*p* < 0.01). In addition, the percentage of girls who experienced menarche before the age of 14 years increased from 76 % in 2001 to 92 % in 2010/2011 (*p* < 0.005). Adolescents whose mothers who had experienced early menarche (HR 1.48, 95 % CI [1.22–1.80]), and adolescents who were overweight (HR 1.24, 95 % CI [1.04–1.49]) were more likely to have experienced menarche. Additionally, underweight adolescents (HR 0.27, 95 % CI [0.12–0.60]) and adolescents who had a mother having late menarche (HR 0.68, 95 % CI [0.59–0.79]) were expected to have late menarche. None of the socioeconomic factors assessed in our study showed an association with age at menarche.

**Conclusions:**

A downward trend in age at menarche was defined in Korean adolescents during the last decade. Furthermore, influences of genetic and nutritional parameters on individual variance in age at menarche were defined.

## Plain English summary

Early menarche has been implicated in adverse health events, including breast cancer, cardiovascular incidents, and mortality. A downward trend in age at menarche has been leveling off in most of industrialized countries in the world over the last few decades. Recently, a trend of earlier pubertal onset in Korean adolescents has been reported. We sought to examine the trend of age at menarche during the last 11 years and associated factors influencing the age at menarche in Korean adolescents. Study subjects were adolescent girls aged 10 to 18 years who participated the Korean National Health and Nutrition Examination Surveys (KNHANES) from 2001 to 2011. Subjects were asked about whether they had experienced menstruation and the year of their menarche using a questionnaire. The mean age at menarche declined from 13.4 years in 2001 to 12.4 years in 2010/2011. Also, the percentage of subjects who experienced menarche before the age of 12 years increased 21.4 % in 2001 to 34.6 % in 2010/2011. Adolescents whose mothers had experienced early menarche and overweight adolescents were more likely to experience earlier menarche. Additionally, adolescents whose mothers had experienced late menarche and underweight adolescents were expected to have later menarche. In conclusion, an ongoing declining trend in age at menarche was noted in Korean adolescents. Nutritional status and genetic factor might be significant factors determining age at menarche. Considering the importance of age at menarche on women’s health and reproductive capacity, continued monitoring of age at menarche and its associated factors is needed in the future.

## Background

Although up to 50–80 % of the variance in human pubertal timing is determined by genetic factors, the substantial decline in the age at menarche between the mid-19th and the mid-20th centuries has been proposed to result from the remarkable refinement of nutrition and human living conditions attained during the process of modern civilization [1, 2].

It is well known that since the 1960s, downward trend in the age at menarche has been substantially slowed or arrested in industrialized countries [3–7]. However, in the last 2 decades, a modest but evident trend of earlier menarche has been reported again in European, American, and Asian countries with a less-developed as well as those with a well-developed industrial base [8–14]. Although childhood and adolescent body weight are well documented as environmental factors with the greatest influence on the age at menarche [4, 10, 15], increased body fat does not completely explain the recent decrease in the age at menarche in girls from well-developed countries [16].

In Korea, two retrospective studies reported downward secular trends in the age at menarche in women born before the mid-1980s [17, 18]. These two studies represented a coherent negative trend until the 1985-year-of-birth cohort; however, they intrinsically included a probability of bias in reporting age at menarche secondary to the long recall interval from time of menarche. Furthermore, no data was available regarding trends in age at menarche in women born after the mid-1980s.

A secular increase in body weight is reported in Korean children and adolescents [19, 20]. In addition, we recently reported a significantly increased annual incidence of central precocious puberty in Korean girls based on 2004–2010 data from the Korean Health Insurance Review Agency [21]. These data suggest an ongoing downward trend in pubertal development in the Korean population.

The objectives of this study were to verify the trend of age at menarche in young Korean women and associated influencing factors.

## Methods

### Study samples

We analyzed data from the Korean National Health and Nutrition Examination Surveys (KNHANES). This is a nationwide, community-based, cross-sectional survey on health and nutritional conditions, designed to represent the non-institutionalized, civilian Korean population. A stratified, multistage probability sampling design was used for the selection of household units. KNHANES consisted of a health behavior (household interviews), nutrition (self-administrated questionnaires), and health examination (standardized physical examinations) surveys. The survey was initially conducted every 3 years by the Korea Institute for Health and Social Affairs; and has, since 2007, been carried out annually by the Division of Chronic Disease Surveillance, Korea Centers for Disease Control and Prevention (KCDCP). The raw data is available at http://knhanes.cdc.go.kr/knhanes/index.do. We conducted an analysis of data from adolescent girls, aged 10 to 18 years, from KNHANES II (2001), III (2005), IV (2007–2009), and V (2010/2011). Subjects were asked whether they had experienced menstruation and the year of their menarche using a self-administered (in girls aged 12 years or older) or parent-answered (in girls aged 11 years or younger) questionnaire. Of the 3409 girls who answered the status of menarche question; 2402 girls reported “experienced menarche” and their age at menarche.

### Predictors of age at menarche

We assessed the associations between age at menarche and genetic, nutritional, and socioeconomic parameters. For the evaluation of genetic contribution, maternal age at menarche was assessed as a categorical variable. This variable was sub-grouped into three categories based on age distribution: younger (<13.0 years), normal (13.0–15.9 years), and older (≥16.0 years). Among the three nutritional parameters, body weight (kg) and height (cm) were evaluated as continuous variables. Also, BMI (kg/m^2^) was evaluated as a categorical variable and stratified into four levels according to the age and sex specific percentiles (%iles) of Korean national reference standards for BMI [22]: underweight (BMI <5^th^ %ile), normal weight (BMI 5^th^–84^th^ %ile), overweight (BMI 85^th^–94^th^ %ile), and obese (BMI >95^th^ %ile). All socioeconomic variables (including household income, parental educational achievement, and type of residential area) were assessed as categorical variables. Household income was categorized into four categories in KNHANES: low, lower middle, upper middle, and high. Completed parental education was classified into five categories: no schooling, elementary school, middle school, high school, and college or university. Type of residence was divided into urban or rural. Specifically, among the 16 districts of South Korea, eight major cities (Seoul, Gyeonggi, Busan, Daegu, Incheon, Gwangju, Daejeoun, and Ulsan) were classified as urban areas and other provinces were classified as rural areas.

### Statistical analysis

Levels of various characteristics and the distribution of covariates between each subgroup were compared using analysis of variance (ANOVA) and chi-squared tests, and data were presented as mean ± standard deviation (SD) or as numbers with percentages. To determine the average age at menarche, means and standard errors (SE) were calculated for age at menarche using Kaplan-Meier survival analysis. The percentage of subjects who had experienced menarche at each age level was compared by Cochran-Armitage test.

Associations between age at menarche and its theoretical predictors were estimated using the Cox proportional hazards model. All variables were initially investigated for univariate associations with age at menarche, and statistical significance was assessed using hazard ratios (HRs) with a 95 % confidence interval. Only variables found to be significantly associated with age at menarche in univariate analyses were adopted for the multivariate regression model to assess independent significance. Statistical analyses were carried out with SAS statistical software, version 9.1 (SAS Institute Inc., Cary, NC, USA). A two-tailed *p* value of <0.05 was considered statistically significant in all analyses.

## Results

### Secular changes in clinical predictors

Participants’ demographic and clinical characteristics are presented in Table 1. Between 2001 and 2010/2011, adolescent participants gained minimal height (*p* < 0.05) without gaining weight; therefore, mean BMI decreased nominally from 20.2 to 20.1 kg/m^2^ (*p* < 0.05). Percentages of participants who were underweight or obese increased slightly, whereas the percentage of participants who were overweight decreased marginally (*p* < 0.05). Regarding household income, proportions of participants in the upper-middle and lower-middle classes increased somewhat, and proportions in high and low classes decreased minimally between 2001 and 2010/2011 (*p* < 0.01).

**Table 1:** Characteristics of participants, girls aged 10 to 18 years in KNHANES II to V

	Total 2001–2011	II 2001	III 2005	IV 2007–2009	V 2010/2011	*p*-value
*N*, total respondents	3409	680	576	1299	854	.0297
*n*, experienced menarche	2402	460	389	926	627	
*n*, before menarche	1007	220	187	373	227	
Age (y)	13.6 ± 2.5	13.7 ± 2.6	13.5 ± 2.5	13.5 ± 2.5	13.7 ± 2.5	.3322
Weight (kg)	48.9 ± 10.8	49.0 ± 10.3	48.8 ± 10.5	48.3 ± 10.9	49.6 ± 11.2	.1024
Height (cm)	155.7 ± 8.5	155.1 ± 8.6	155.8 ± 8.3	155.5 ± 8.7	156.5 ± 8.3	.0204
BMI (kg/m^2^)	20.0 ± 3.3	20.2 ± 3.2	20.0 ± 3.2	19.8 ± 3.3	20.1 ± 3.5	.0193
BMI percentile (%)						
Underweight	154 (5.1)	22 (3.9)	22 (5.5)	72 (6.0)	38 (4.5)	.0411
Normal	2422 (80.1)	448 (78.7)	331 (83.0)	965 (80.2)	678 (79.4)	
Overweight	298 (9.9)	69 (12.1)	26 (6.5)	118 (9.8)	85 (10.0)	
Obese	151 (5.0)	30 (5.3)	20 (5.0)	48 (4.0)	53 (6.2)	
Household income percentile (%)						
High	980 (29.7)	211 (33.7)	146 (25.9)	387 (30.5)	236 (28.1)	.0030
Upper middle	1014 (30.7)	163 (26.0)	175 (31.0)	411 (32.4)	265 (31.6)	
Lower middle	853 (25.8)	152 (24.2)	149 (26.4)	327 (25.8)	225 (26.8)	
Low	454 (13.8)	101 (16.1)	94 (16.7)	145 (11.4)	114 (13.6)	
Place of residence percentile (%)						
Urban	2327 (68.3)	466 (68.5)	394 (68.4)	865 (66.6)	602 (70.5)	.3001
Rural	1082 (31.7)	214 (31.5)	182 (31.6)	434 (33.4)	252 (29.5)	
Maternal age at menarche percentile (%)						
Younger (<13.0 y)	177 (9.1)	19 (3.2)	26 (5.5)	30 (11.7)	102 (16.2)	<.0001
Normal (13.0–15.9 y)	1282 (66.2)	324 (56.5)	345 (72.5)	194 (75.5)	419 (66.6)	
Older (≥16.0 y)	479 (24.7)	233 (40.5)	105 (22.1)	33 (12.8)	108 (17.2)	

Data are expressed as mean ± SD or number of participants (%)

*Abbreviations*: *KNHANES* Korean National Health and Nutrition Examination Survey, *BMI* body mass index

### Age at menarche and its trend

The overall mean age at menarche was estimated at 12.7 years (Table 2). The mean age at menarche decreased between 2001 and 2010/2011, from 13.4 years in 2001 to 12.4 years in 2010/2011 (*p* < 0.0001). Of note, the percentage of girls who experienced menarche before the age of 12 years (*p* < 0.01), 13 years (*p* < 0.005), and 14 years (*p* < 0.005) increased significantly from 21.4, 42.3, and 76.0 % in 2001 to 34.6, 62.2, and 92.0 % in 2010/2011, respectively. An increasing trend in the percentage of girls who experienced menarche before the age of 11 and 15 years was also suggested. Overall, 95.8 % of girls had experienced menarche before 15.0 years of age in these study participants.

**Table 2:** Average age at menarche and percentage of girls who had experienced menarche by age

	Total 2001–2011		II 2001		III 2005		IV 2007–2009		V 2010/2011		*p*-value
Average age at Menarche											
Mean SE	12.71 ± 0.03		13.40 ± 0.06		13.08 ± 0.06		12.38 ± 0.04		12.38 ± 0.05		<0.0001
Number of total respondents											
	3409		680		576		1299		854		
Number of respondents experienced menarche											
	2402		460		389		926		627		
Percentage of girls who experienced menarche											
Age (y)	*n*	%	*n*	%	*n*	%	*n*	%	*n*	%	
10.0–10.9	461	8.0	91	8.8	79	5.1	174	7.5	117	10.3	0.6429
11.0–11.9	446	26.7	84	21.4	76	15.8	176	29.0	110	34.6	0.0084
12.0–12.9	410	60.0	78	42.3	68	63.2	174	65.5	90	62.2	0.0036
13.0–13.9	422	85.3	79	76.0	82	80.5	148	87.8	113	92.0	0.0018
14.0–14.9	403	95.8	76	94.7	68	95.6	161	95.7	98	96.9	0.4384
15.0–15.9	347	98.0	75	97.3	59	96.6	133	100.0	80	96.3	0.4801
16.0–16.9	331	98.8	65	98.5	53	98.1	125	99.2	88	98.9	0.6114
17.0–17.9	341	99.4	77	100.0	54	98.2	114	99.1	96	100.0	0.9318
18.0–18.9	248	99.2	55	100.0	37	97.3	94	98.9	62	100.0	0.9071

Data are expressed as mean ± SE

*n*: the total number of girls at each age who had answered the status of menarche

%: the percentage of girls who had experienced menarche among girls who had answered the status of menarche question

### Factors influencing age at menarche

The results of the univariate Cox proportional hazard models are shown in Table 3. Higher body weight was associated with odds of having experienced menarche (HR 1.01, 95 % CI [1.01–1.02]). In the analysis of categorical variables; younger maternal age at menarche (HR 1.51, 95 % CI [1.26–1.83]), obesity (HR 1.26, 95 % CI [1.06–1.50]), being overweight (HR 1.23, 95 % CI [1.08–1.40]), high household income (HR 1.21, 95 % CI [1.06–1.38]), and upper-middle household income (HR 1.16, 95 % CI [1.02–1.32]) were associated with having experienced menarche. Conversely, being underweight (HR 0.33, 95 % CI [0.19–0.55]) and older maternal age at menarche (HR 0.71, 95 % CI [0.62–0.80]) showed correlations with later occurrence of menarche. Participant’s height, place of residence, and parental educational levels failed to show significant correlations with age at menarche (*p* > 0.05); therefore, they were not included in multivariate analyses.

**Table 3:** Univariate Cox proportional hazard model of age at menarche

Variables	Hazard Ratio (95 % CI)	*p*-value
Bodyweight (kg)	1.013 (1.008–1.017)	<.0001
Height (cm)	1.003 (0.996–1.010)	.4293
Maternal age at menarche (y)		
Younger (<13.0)	1.514 (1.256–1.826)	<.0001
Normal (13.0–15.9)	1.00	
Older (≥16.0)	0.707 (0.622–0.804)	<.0001
BMI (kg/m^2^)		
Underweight	0.327 (0.193–0.554)	<.0001
Normal	1.00	
Overweight	1.227 (1.076–1.400)	.0023
Obese	1.258 (1.058–1.495)	.0093
Household income		
Low	1.00	
Lower middle	1.117 (0.978–1.276)	.1030
Upper middle	1.159 (1.018–1.320)	.0259
High	1.207 (1.059–1.378)	.0047
Paternal education level		
No schooling	1.00	
Elementary school	1.188 (0.571–2.474)	.6446
Middle school	0.997 (0.487–2.040)	.9924
High school	1.266 (0.628–2.552)	.5093
College or university	1.463 (0.752–2.950)	.2880
Maternal education level		
No schooling	1.00	
Elementary school	0.974 (0.504–1.880)	.9363
Middle school	0.895 (0.471–1.701)	.7351
High school	1.096 (0.585–2.053)	.7740
College or university	1.370 (0.729–2.577)	.3280
Place of residence		
Rural	1.00	
Urban	1.039 (0.954–1.131)	.3804

**Fig. 1:**
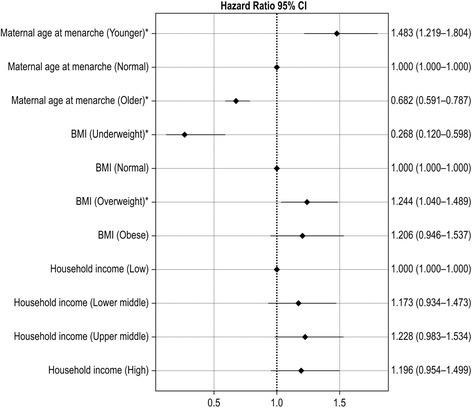
Multivariate Cox proportional hazards model of age at menarche

Figure 1 illustrates the outcome of the multivariate analyses. Younger maternal age at menarche (HR 1.48, 95 % [CI 1.22–1.80]) and being overweight (HR 1.24, 95 % [CI 1.04–1.49]) were consistently associated with occurrence of menarche. In addition, older maternal age at menarche (HR 0.68, 96 % CI [0.59–0.79]) and being underweight (HR 0.27, 95 % CI [0.12–0.60]) were negatively associated with occurrence of menarche. However, the association between age at menarche and obesity was no longer statistically significant when analyzed in a multivariate regression model. This was also the case for the association with age at menarche and household income.

## Discussion

One of the objectives of our study was to document the age at menarche over time in the Korean adolescent population. During the past decade, several studies from various countries have updated the average age at menarche and reported a range between 12.0 and 13.0 years [11–13, 23–25]. In particular, two recent Asian studies reported the average age at menarche as 12.27 and 12.41 years [11, 25]. The availability of data in Korea has been sparse in recent decades; however, Cho et al. [18] reported a mean age at menarche in Korean girls of 13.1 years after analyzing data from KNHANES III (2005). Based on our analyses using KNHANES II (2001), III (2005), IV (2007–2009), and V (2010/2011), the overall mean age at menarche in the last decade was 12.7 years.

Until now, studies from different countries have reported a continuous downward trend in age at menarche in the last two decades [4, 7, 9, 10, 13, 14]. In Korea, two well-organized previous studies reported a downward secular trend in age at menarche, −0.64 to −0.68 years per decade, in women born between 1920 and 1985 [17, 18]. However, no data was available regarding the recent trend of age at menarche in Korean population born after the mid-1980s. Of note, the percentage of girls who experienced menarche at each age in our study demonstrated earlier trend of age at menarche in the participants born between 1983 and 2001 (Table 2). This suggests a certain degree of ongoing downward trend of age at menarche in Korean girls born after the mid-1980s.

Age at menarche is known to be influenced by body weight [26], BMI [10, 23, 25, 27, 28], socioeconomic status [24, 29, 30], maternal age at menarche [7, 31], and ethnicity [3, 5, 8, 10, 32]. In addition, the influence of childhood and adolescent body fat on the timing of sexual maturation in females is well documented [3, 4, 10, 15, 33, 34]. Recently, one study group reported a specific genetic variant that contributed to early menarche in a cohort of obese girls [35]. Moreover, other factors, including prenatal nutrition, dietary component, and exposure to endocrine disruptors, have been suggested as likely contributors to the secular trend in earlier pubertal development [36]. Accordingly, we have previously reported an association between high serum isoflavone concentrations and the risk of precocious puberty in Korean girls [37].

The multivariate Cox proportional hazard model in our present study demonstrated a clear influence of maternal age at menarche on individual age at menarche (Fig. 1). In terms of BMI, both being underweight and being overweight were associated with age at menarche; however, the association between age at menarche and obesity lost statistical significance when analyzed in a multivariate regression model. As reported by another group [16], this suggests a limited influence of BMI on age at menarche in high-BMI groups. This phenomenon can be explained by hyperandrogenism in obese girls [38]. Obesity in girls is frequently associated with hyperandrogenism; some girls with hyperandrogenism have an increased risk of adolescent polycystic ovary syndrome (PCOS) [38], which may cause delayed menarche in pubertal girls [39]. Additionally, socioeconomic factors assessed in our study failed to demonstrate an association with age at menarche in Korean adolescents. This finding supports the notion that the socioeconomic factor is not an independent predictor of age at menarche in well-developed countries [14, 40] or urbanized populations [6].

Although some previous Korean studies reported a secular increase in body weight in Korean girls until 2005 [19, 20], there were no corresponding changes in mean values of body weight, height, BMI, or proportions of BMI subgroups in our adolescent participants between 2001 and 2010/2011. Consequently, a decline in age at menarche accordingly in this study could not be suitably explained by changes in nutritional parameters. Considering negligible changes in nutritional parameters, future research on the cause of downward secular trend of age at menarche should focus on other factors including perinatal nutrition, dietary components, physical inactivity and exposure to endocrine disruptors.

In this study, it was noted that the proportion of participants who start to menstruate younger than 15.0 years was 95.8 % (Table 2). This value was highly correlated with a 2 SD score above the mean; therefore, this finding supports the rationale of using an age limit of 15 years in defining primary amenorrhea in the Korean adolescent population.

A major strength of this study was the use of the most recent, large-scale national samples designed to represent the non-institutionalized, civilian Korean population. A major limitation of our study is the use of retrospective data on age at menarche. Although we utilized Kaplan-Meier survival analyses, we could not obtain precise median and SD scores of age at menarche. In addition, this study analyzed a series of cross-sectional data sets. While the Cox proportional hazards model evaluated associations between age at menarche and related factors, no assumptions of causal inference can be drawn from this study.

## Conclusions

This study provides an update of the latest trend in age at menarche in Korean adolescents and demonstrates a continuous downward secular trend during the last decade. In addition, it defines the influence of genetic and nutritional parameters on individual variance in age at menarche. Future research on the cause of the downward secular trend in age at menarche is suggested.

## Abbreviations

ANOVA: Analysis of variance
BMI: Body mass index
HR: Hazard ratios
KCDCP: Korea Centers for Disease Control and Prevention
KNHANES: Korean National Health and Nutrition Examination Surveys
PCOS: Polycystic ovary syndrome
SD: Standard deviation
SE: Standard errors
